# Profiles of Global Gene Expression in Ionizing-Radiation–Damaged Human Diploid Fibroblasts Reveal Synchronization behind the G_1_ Checkpoint in a G_0_-like State of Quiescence

**DOI:** 10.1289/ehp.8026

**Published:** 2005-12-19

**Authors:** Tong Zhou, Jeff W. Chou, Dennis A. Simpson, Yingchun Zhou, Thomas E. Mullen, Margarida Medeiros, Pierre R. Bushel, Richard S. Paules, Xuebin Yang, Patrick Hurban, Edward K. Lobenhofer, William K. Kaufmann

**Affiliations:** 1 Department of Pathology and Laboratory Medicine, Center for Environmental Health and Susceptibility, and Lineberger Comprehensive Cancer Center, University of North Carolina at Chapel Hill, Chapel Hill, North Carolina, USA; 2 National Institute of Environmental Health Sciences, National Institutes of Health, Department of Health and Human Services, Research Triangle Park, North Carolina, USA; 3 Paradigm Array Labs, Icoria Inc., Research Triangle Park, North Carolina, USA

**Keywords:** cell cycle checkpoints, DNA damage, gene expression, human fibroblasts, ionizing radiation, microarray

## Abstract

Cell cycle arrest and stereotypic transcriptional responses to DNA damage induced by ionizing radiation (IR) were quantified in telomerase-expressing human diploid fibroblasts. Analysis of cytotoxicity demonstrated that 1.5 Gy IR inactivated colony formation by 40–45% in three fibroblast lines; this dose was used in all subsequent analyses. Fibroblasts exhibited > 90% arrest of progression from G_2_ to M at 2 hr post-IR and a similarly severe arrest of progression from G_1_ to S at 6 and 12 hr post-IR. Normal rates of DNA synthesis and mitosis 6 and 12 hr post-IR caused the S and M compartments to empty by > 70% at 24 hr. Global gene expression was analyzed in IR-treated cells. A microarray analysis algorithm, EPIG, identified nine IR-responsive patterns of gene expression that were common to the three fibroblast lines, including a dominant p53-dependent G_1_ checkpoint response. Many p53 target genes, such as *CDKN1A*, *GADD45*, *BTG2*, and *PLK3*, were significantly up-regulated at 2 hr post-IR. Many genes whose expression is regulated by E2F family transcription factors, including *CDK2*, *CCNE1*, *CDC6*, *CDC2*, *MCM2*, were significantly down-regulated at 24 hr post-IR. Numerous genes that participate in DNA metabolism were also markedly repressed in arrested fibroblasts apparently as a result of cell synchronization behind the G_1_ checkpoint. However, cluster and principal component analyses of gene expression revealed a profile 24 hr post-IR with similarity to that of G_0_ growth quiescence. The results reveal a highly stereotypic pattern of response to IR in human diploid fibroblasts that reflects primarily synchronization behind the G_1_ checkpoint but with prominent induction of additional markers of G_0_ quiescence such as *GAS1*.

Exposure to ionizing radiation (IR) produces several forms of cellular DNA damage, including formation of uracil, apurinic/apyrimidinic sites, 8-oxoguanine, single-strand breaks, and double-strand breaks (Heinloth et al. 2003; Slupphaug et al. 2003). Cell cycle checkpoint responses to IR-induced DNA damage employ a complex network of gene products that cooperate to delay progression through the interphase compartments of the cell cycle and enhance repair of damaged DNA (Fornace et al. 2002). When DNA damage is irreparable, checkpoints also inactivate clonogenic survival by permanent cell cycle arrest or apoptosis. Posttranslational modifications of proteins in the ATM/ATR (ataxia telangiectasia mutated/ATM- and Rad3-related) CHK1/CHK2 (checkpoint kinase 1/checkpoint kinase 2), and p53 signaling pathways have been well studied in response to IR-induced DNA damage (Abraham 2003; Bartek and Lukas 2001; Falck et al. 2001). Several studies have also shown that global gene expression, including expression of many cell cycle–regulated genes, is markedly affected by IR (Amundson et al. 2001; Bishay et al. 2000; Cheung et al. 2003; Heinloth et al. 2003). The transcriptional regulation of cell cycle–regulated genes may be closely related to checkpoint functions upon DNA damage. Changes in gene expression may be a mechanism for initiation of cell cycle arrest or a consequence of cell synchronization. The relationships between cell cycle checkpoint function and transcriptional regulation of gene expression have not been systematically studied in normal human fibroblast lines.

In the present study, the changes in global gene expression that occur in normal human fibroblasts in response to IR-induced DNA damage were determined using 20K Agilent human 1A microarrays. Three different lines were analyzed to identify stereotypic patterns of response to IR that are common to human fibroblasts. Gene expression profiles were analyzed using a method called “extracting microarray gene expression patterns and identifying biologically significant genes” (EPIG; Chou JW, Zhou T, Paules RS, Kaufmann WK, Bushel PR, unpublished method). EPIG extracts significant patterns by calculating the correlations of gene expression and then identifies significant genes based on their correlations with a specific pattern, the signal-to-noise ratio, and the magnitude of change. Nine distinct patterns including 1,811 IR-responsive genes were observed. Congruent analyses of gene expression and checkpoint-dependent delays in progression through the cell cycle revealed a complex integration of networks of DNA damage response and cell division. A dominant p53-dependent G_1_ checkpoint response to IR was recognized that led to repression of approximately 900 growth-related transcripts as the cell culture was depleted of S- and M-phase cells. However, fibroblasts at 24 hr post-IR showed greater similarity to G_0_- than to G_1_-synchronized fibroblasts, indicating for the first time that the IR-induced growth arrest also included induction of quiescence-associated transcripts.

## Materials and Methods

### Cell lines and culture.

Normal human fibroblast strains, NHF1, NHF3, and NHF10, were derived from neonatal foreskins and established in secondary culture according to established methods (Maher et al. 1981). Immortal cell lines were obtained by ectopic expression of human telomerase (hTERT), as previously described (Deming et al. 2001; Heffernan et al. 2002). Fibroblasts were cultured in Dulbecco’s modified Eagle’s medium (Invitrogen, Carlsbad, CA) supplemented with 2 mM l-glutamine (Invitrogen) and 10% fetal bovine serum (Sigma Chemical Co., St. Louis, MO). All cell lines were maintained at 37°C in a humidified atmosphere of 5% CO_2_ and were tested and shown to be free of mycoplasma contamination using a commercial kit (Gen-Probe, San Diego, CA). Cytogenetic analysis established that all three fibroblast lines were 46XY with normal chromosome numbers and structure (data not shown).

### Cell irradiation.

We exposed cells to IR in culture medium using a cesium-137 source (Gammacel40; Atomic Energy of Canada Ltd., Ottawa, Canada) at a dose rate of 0.84 Gy/min. Sham-treated controls were subjected to the same movements in and out of incubators as were irradiated cells.

### Clonogenic survival assay.

Clonogenic survival was measured in logarithmically growing NHF1, NHF3, NHF10 fibroblasts, plated at 400–500 cells per 100-mm–diameter dish and incubated for 8 hr before exposure to IR (three dishes per dose). Cells were cultured for 2 weeks, with medium changed twice a week. Colonies were fixed and stained with a solution of 40% methanol and 0.05% crystal violet. Colonies with ≥ 50 cells were counted. The relative colony-forming efficiency of IR-treated cells was expressed as a fraction of sham-treated controls.

### Cell cycle checkpoint assays.

We quantified G_1_ checkpoint function using flow cytometry to determine the IR-induced reduction in the percentage of bromodeoxyuridine (BrdU)-labeled cells in the first half of S phase 6–8 hr after 1.5 Gy (Doherty et al. 2003). G_2_ checkpoint function was quantified by using flow cytometry to determine the IR-induced reduction in the percentage of phosphohistone H3–labeled mitotic cells 2 hr after 1.5 Gy (Doherty et al. 2003). To determine the percentage changes in the fractions of cells in various cell cycle compartments with time post-IR, we incubated cells with BrdU for 2 hr beginning 2, 6, 12, and 24 hr after 1.5 Gy or sham treatment. Cells were harvested and analyzed for BrdU incorporation and expression of phosphohistone H3. Summit flow cytometry analysis software (Dako Cytomation, Fort Collins, CO) was used to quantify the numbers of unlabeled cells with 2N (G_0_/G_1_) and 4N DNA content (G_2_), BrdU-labeled cells with 2–4N DNA content (S), and 4N cells with high phosphohistone H3 (M).

### Cell synchronization.

NHF1, NHF3, and NHF10 fibroblasts were synchronized as previously described (Cordeiro-Stone et al. 1986). Briefly, we plated cells at a density of 1.3 × 10^4^/cm^2^ and allowed them to grow for 8 days to confluence arrest (G_0_ phase). Cells were fed on days 3 and 5 postseeding. We trypsinized, reseeded, and incubated cells for 8 hr in fresh medium to allow them to reach G_1_ phase. Then, 1 × 107 cells were harvested at each growth phase for RNA isolation. Flow cytometric analysis indicated that > 90% of cells were synchronized to G_0_ by confluence arrest, and 8 hr after release from confluence and reseeding in serum-containing medium, > 90% remained with 2N DNA content (G_1_) (Unsal-Kacmaz et al. 2005). Using this synchronization method, cells began to enter S phase 12 hr after release from confluence arrest (Cordeiro-Stone et al. 1986; Dulic et al. 1994).

### Oligo DNA microarray.

Logarithmically growing NHF1, NHF3, and NHF10 cells were treated with 1.5 Gy IR, or sham-treated, and harvested at 2, 6, or 24 hr after the treatment. Total RNA was isolated using an RNeasy kit (Qiagen Inc., Valencia, CA). The quality of all RNA samples was confirmed using an Agilent 2100 bioanalyzer. Microarray analysis was then performed. Briefly, 1 μg of sample RNA and global reference RNA (Stratagene, La Jolla, CA) were converted to cDNA with reverse transcriptase and then amplified using T7 RNA polymerase while labeling with either cyanine 3 deoxyuridine triphosphate (Cy3-dUTP) or Cy5-dUTP (Low RNA Input Linear Amplification Kit, Agilent Techologies). The quality of each labeled cRNA was evaluated using an Agilent 2100 bioanalyzer before hybridization; 750 ng of Cy3- and Cy5-labeled cRNA were used in the hybridization. The labeled cRNA from sham-or IR-treated samples was hybridized with the labeled global reference cRNA on an Agilent 22K human 1A array (Agilent Techologies) in a hybridization oven (model 400, 1040-60-1AG; Robbins Scientific, Sunnyvale, CA) at 60°C for 17 hr. Hybridization of sample RNA against reference RNA was done twice with dye swap. After hybridization, we scanned the arrays using the Agilent DNA Microarray Scanner with SureScan Technology; microarray images were analyzed using Agilent Feature Extraction software (version 7.1; Agilent Techologies). The gene expression level was presented as the ratio of sample intensity against reference intensity.

### Microarray data analysis.

The extracted intensity data from each array were preprocessed, which included array-based systematic variation normalization (Chou et al. 2005), profile-based dye-swap correction, and biological reference state alignment (Chou JW, Zhou T, Raules RS, Kaufmann WK, Bushel PR, unpublished data). In this data set, the sham-treated condition was used as a reference state. The average of the dye-swapped pair of sham-treated control arrays was aligned to log zero as a baseline, with the IR-treated samples adjusted by the same amount. Through signal-to-noise ratio evaluation applied to each correlation local cluster, EPIG extracted a set of discrete gene expression patterns. Each pattern represented a set of co-expressed genes. EPIG used the profile’s signal magnitude and signal-to-noise ratio to identify biologically significant genes and categorized them within patterns according to their correlation *r*-values. (Chou Chou JW, Zhou T, Raules RS, Kaufmann WK, Bushel PR, unpublished data). Two-way clustering heat maps were made by using Cluster and Treeview software (Eisen et al. 1998). Three-dimensional principal component analysis (PCA) was done using EPIG. Categories of genes that were overrepresented in a selected gene list, compared with what was represented in the microarray, were analyzed using Expression Analysis Systematic Explorer (EASE; http://david.niaid.nih.gov/david/ease.htm). Such overrepresented categories represent biological “themes” of a given list (Hawes et al. 2005).

## Results

### Dose-dependent inactivation of clonogenic survival by IR.

**Figure 1:**
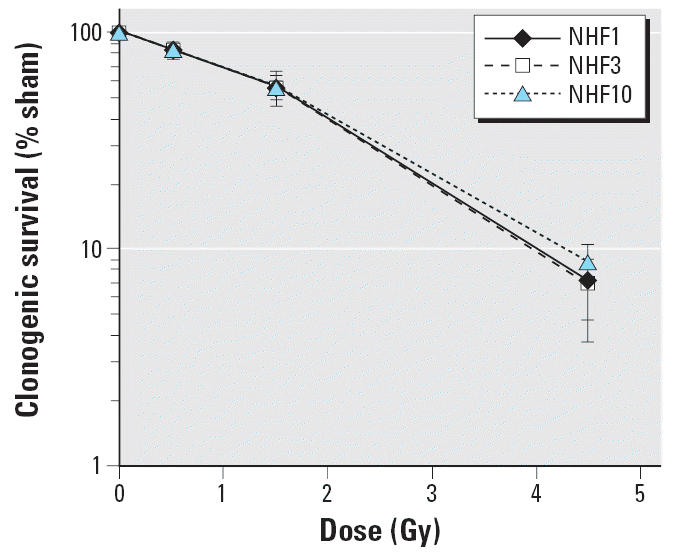
Inactivation of clonogenic survival by IR. Three human diploid fibroblast lines were irradiated with 0, 0.5, 1.5, and 4.5 Gy IR, and colonies were counted after a 14-day incubation. Results show the mean relative colony formation in irradiated cultures (mean ± SD, *n* = 3).

Treatment with IR inhibited single-cell colony formation in three diploid human fibroblast lines, NHF1, NHF3, and NHF10, with similar dose kinetics (Figure 1). A shoulder on survival curves was apparent below the 1.5-Gy dose. The slope of the curves between the 1.5- and 4.5-Gy doses approximated a D0 (lethel dose) dose of about 1.5 Gy similar to D0 (mean lethal dose) values previously recorded for IR-treated normal human fibroblasts (Arlett et al. 1988). The 1.5-Gy dose reduced colony formation in fibroblasts by 40–45% relative to sham-treated controls and was selected for further analysis of cell cycle checkpoint responses and changes in gene expression.

### IR-induced cell cycle checkpoint responses synchronize cell division.

**Figure 2:**
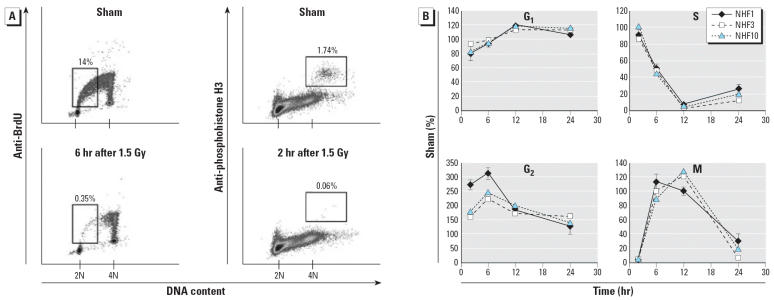
IR-induced G_1_ and G_2_ checkpoint functions in diploid human fibroblasts. (*A*) Representative profiles of flow cytometric analyses of BrdU incorporation 6–8 hr after 1.5 Gy IR or sham treatment of NHF1 and anti-phosphohistone H3 antibody labeling 2 hr after 1.5-Gy or sham treatment of NHF1. (*B*) Dynamic changes in cell cycle compartments over time. The percentages of cells in G_0_/G_1_, S, G_2_, and M phases were determined from flow cytometric profiles. The values depicted for each fibroblast line are the mean percentages of IR-treated cells in each cycle phase compartment expressed as a percentage of the appropriate sham-treated controls (*n* = 2–3). Error bars indicate SD.

We quantified the IR-induced G_1_ checkpoint by measuring the incorporation of bromodeoxyuridine 6–8 hr after 1.5-Gy or sham treatment (Figure 2A, left). IR-treated fibroblasts displayed a severe reduction in the fraction of BrdU-labeled S-phase cells with 2–3N DNA content (first half of S) due to ATM- and p53-dependent G_1_ arrest (Kaufmann et al. 2003). The three fibroblast lines exhibited > 93% G_1_ arrest (Table 1). The IR-induced G_2_ checkpoint was quantified by measuring mitosis-specific phosphohistone H3 immunostaining 2 hr post-IR or sham treatment (Figure 2A, right). IR-treated fibroblasts displayed a severe reduction in the fraction of mitotic cells because of ATM-dependent G_2_ arrest. The three fibroblast lines exhibited > 94% G_2_ arrest (Table 1).

Dynamic changes in DNA content, DNA synthesis, and mitosis were charted 2–24 hr post-IR (Figure 2B). The three cell lines showed very similar responses to IR. The percentage of cells with 2N DNA that did not incorporate BrdU (G_1_) showed a small decline at 2 hr post-IR and then rose to a plateau 10% above control by 12 hr. S-phase cells with 2–4N DNA content and labeled with BrdU declined between 6 and 12 hr post-IR to a nadir at < 5% of control, then recovered by 24 hr to 10–30% of the sham-treated control. There was a rapid post-IR accumulation of 4N cells with no BrdU incorporation (predominantly G_2_), which peaked at 6 hr and then declined to near control levels by 24 hr. Mitosis was severely inhibited 2 hr post-IR and then recovered to control levels at 6 and 12 hr before falling again at 24 hr to < 25% of control. The recovery of mitotic cells 6 and 12 hr post-IR and the decline in G_2_ cells 6–24 hr post-IR indicate that the G_2_ checkpoint response to IR was a transient arrest that was largely reversed by 6 hr. The severe (70–90%) reduction in Sand M-phase cells 24 hr post-IR is consistent with synchronization of fibroblasts behind a persistent G_1_ checkpoint response.

### Profiles of gene expression in response to IR in normal human fibroblasts.

**Figure 3:**
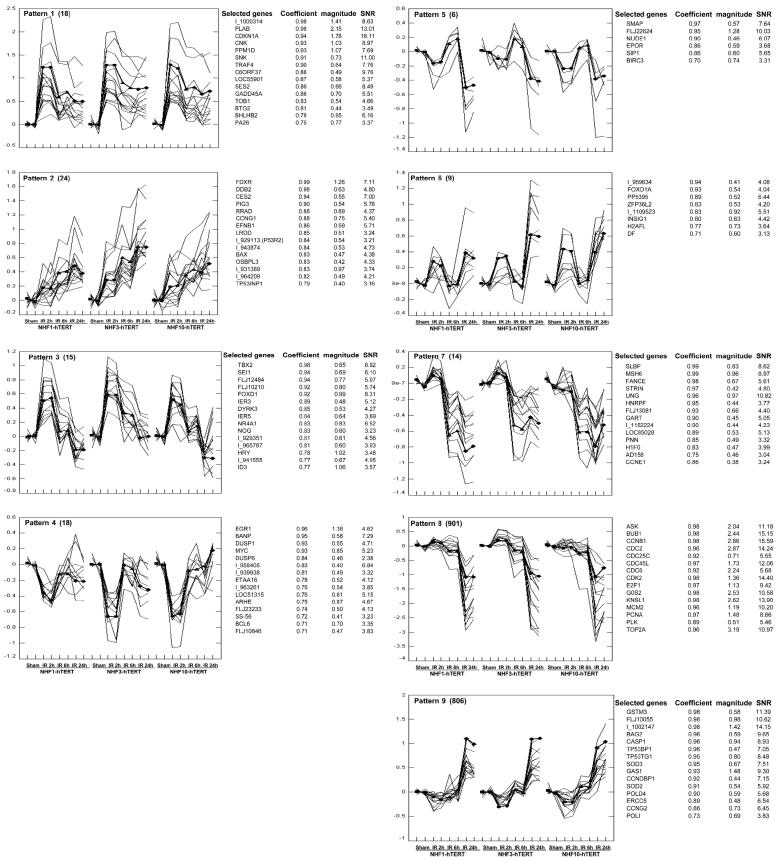
Patterns of gene expression in the three fibroblast lines NHF1, NHF3, and NHF10 in response to IR-induced DNA damage. Nine different DNA-damage–responsive patterns of gene expression were extracted using EPIG, each pattern representing a group of genes that were expressed in the same way upon DNA damage. In each panel (patterns 1–9) the numbers of genes in each pattern are shown in parentheses. For each cell line, the log_2_ ratios of sample RNA against reference RNA are indicated at each time point (IR 2 hr, IR 6 hr, IR 24 hr) with the sham-treated controls adjusted to zero and with both dye-flip replicates shown. The thick lines show the average response to IR of genes in the pattern for each of the fibroblast lines, and the thin lines show the responses of the selected genes listed to the right. For the genes listed, the coefficient of correlation with the average pattern, the magnitude of change in gene expression (log_2_), and the signal-to-noise ratio (SNR) are given.

We used Agilent human 1A arrays (22K) to monitor gene expression post-IR in the three different fibroblast lines. Fibroblasts were harvested 2, 6, and 24 hr after 1.5 Gy, which are times of maximal initial G_2_ arrest with minimal reduction in S phase, maximal G_1_ arrest with recovery of mitosis, and sustained G_1_ arrest with depletion of S phase and mitosis, respectively. Controls were harvested 6 hr after sham treatment. Gene expression profiles obtained from microarray data included 24 arrays. Each cell line (NHF1, NHF3, and NHF10) had four treatment states (sham and 2, 6, or 24 hr after the 1.5 Gy IR) and dye-swapped pairs for each treatment. EPIG extracted nine patterns of change in gene expression and identified a total of 1,811 genes as significantly altered in response to IR (Figure 3). The numbers of genes in each pattern varied from several to several hundreds, and about one-third of the 1,811 selected genes were expressed sequence tags (ESTs). Pattern 1 included 18 genes that were highly induced at 2 hr, then declined modestly through 24 hr. This pattern included prototypical p53-target genes that mainly contribute to initiation and maintenance of G_1_ arrest through inhibition of cyclin-dependent kinases. Pattern 2 included 24 genes that were progressively induced from 2 to 24 hr. These genes also are known to be induced by p53-dependent signaling. *CCNG1* may contribute to recovery of DNA synthesis through attenuation of p53 signaling (Ohtsuka et al. 2004). Pattern 3 included 15 genes that were induced only at 2 hr, including immediate early-response genes *IER3* and *IER5* and a *p16* (*INK4A*) antagonist *SEL1*. Pattern 4 included 18 genes that were repressed only at 2 hr. Repression of *MYC* and the early growth-response gene *EGR1* in this group may further negatively regulate E2F1 and its target gene expression. Pattern 5 included 6 genes that were induced modestly at 6 hr but repressed at 24 hr. Pattern 6 included 9 genes that were induced at 2 and 24 hr but not at 6 hr. Pattern 7 included 14 genes that were highly repressed at 6 and 24 hr, of which *CCNE1* (*cyclin E*) repression is an important indicator of G_1_ arrest starting at 6 hr. Several DNA repair genes, such as *MSH2*, *FANCE*, and *UNG*, were in this group. Pattern 8 was composed of 901 genes that were repressed moderately at 6 hr but highly repressed at 24 hr, including many genes whose products participate in various DNA metabolic events during the cell division cycle, such as *ASK*, *CCNA2*, *CCNB1*, *CCNB2*, *CDK2*, *CDC2*, *CDC6*, *CDC45L*, *CDC7L*, *MCMs*, *RFC* subunits, and *TOP2A* (Mendez and Stillman 2000; Stillman 1996). Several DNA repair genes, *MSH2*, *RAD18*, *RAD51*, *RAD54*, *XRCC1*, *XRCC4*, and *XRCC5*, and two apoptosis inducers, *CASP3* and *HCS*, were also in this category. Pattern 9 included 806 genes that were induced at 24 hr, including *CCNDBP1* (a negative regulator of E2F1), the stress-response genes *GSTM3* and *SOD3*, and p53-dependent genes (*TP53BP1* and *TP53TG1*). A G_0_-specific gene *GAS1* was highly induced at 24 hr.

Gene Ontology (http://david.niaid.nih.gov/david/ease.htm) analysis of the 1,811 genes using EASE revealed that more than 30 biological processes were overrepresented in response to IR-induced DNA damage (Table 2). Categories in which the proportion of IR-responsive genes exceeded expectation based on chance included cell cycle, cell proliferation, DNA and RNA metabolism, M phase of mitotic cell cycle, S phase of mitotic cell cycle, response to DNA damage stimulus, DNA repair, and cell-cycle checkpoint (Table 2). When EASE analysis was focused on each pattern, genes in pattern 1 indicated negative regulation of cell proliferation and cell cycle arrest (Table 3), genes in pattern 8 showed similar categories as analyzed in the entire 1,811 gene list, and genes in pattern 9 showed only several categories that were not obviously related to DNA damage response, such as extracellular matrix, calcium ion binding, and antigen processing. Patterns 2–7 did not display overrepresentation of specific gene categories.

### Global gene expression patterns in fibroblasts reveal individual genetic background, previous IR treatment, and IR-induced G_0_ quiescence.

Many genes display cell cycle–dependent changes in expression, and it has been demonstrated that IR treatment can induce a senescence-like permanent G_1_ arrest in fibroblasts, even after a low dose of 1 Gy (Di Leonardo et al. 1994). We were interested in determining whether the IR-induced changes in gene expression reflected a pattern of G_1_ arrest. Cells were synchronized to G_0_ by growth to confluence and cells were synchronized to G_1_ by replating G_0_ quiescent cells at lower cell density in serum-containing medium with harvest at 8 hr after replating. Previous studies using this method indicated that G_0_-synchronized cells expressed low levels of cyclins A and D1 and hypophosphorylated retinoblastoma (RB), whereas G_1_-synchronized cells expressed increasing levels of cyclins A and D_1_ with hyperphosphorylated RB (Dulic et al. 1994).

**Figure 4:**
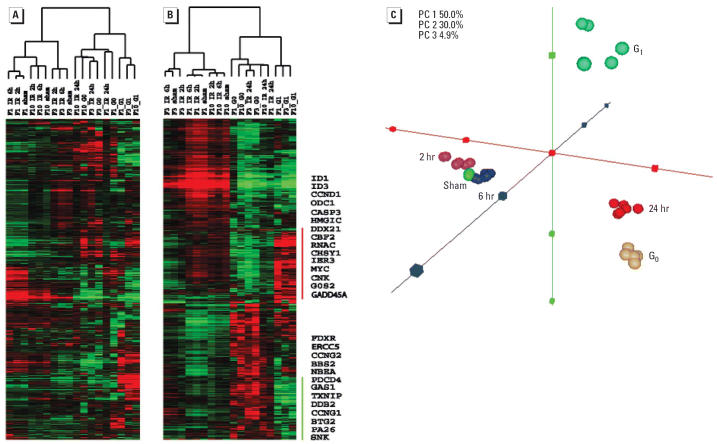
Comparison of gene expression patterns between G_0_- and G_1_-synchronized cells, and IR- or sham-treated cells. (*A*) Unsupervised hierarchical cluster analysis was done by using the 16,930 genes listed in the Agilent human 1A array. Before clustering, the mean center of genes was repeated 5 times, and an uncentered correlation was used for the similarity metric calculation. (*B*) Supervised hierarchical cluster analysis was done using the 1,811 genes significantly changed in response to IR. To the right of the cluster are listed selected genes that show similar expression patterns in G_0_ and 24-hr post-IR samples but a different pattern in G_1_ cells. (*C*) PCA analysis using 1,811 significant genes showed that 24-hr post-IR patterns were very close but did not overlap with G_0_ patterns, and 24-hr post-IR and G_0_ patterns were far from G_1_ patterns.

Unsupervised hierarchical cluster analysis of all genes in the arrays, totaling 16,390 single clones, was performed using data from sham-or IR-treated cells and cells synchronized to G_0_ or G_1_ (Figure 4A). Two main clusters were identified that represent asynchronous and synchronous cell populations. Consistently, the sham, 2-hr IR, and 6-hr IR samples from the same cell line were clustered together in the asynchronous group, demonstrating that gene expression was closely correlated with individual genetic background, and changes in gene expression 2 and 6 hr post-IR did not override basal differences due to interindividual variation. However, all three 24-hr IR samples, despite interindividual differences, were clustered with the synchronized cells. Moreover, the patterns of gene expression 24 hr post-IR were clustered with synchronized G_0_ cells.

We performed a similar analysis using 1,811 genes that were selected by EPIG as showing significant response to IR-induced DNA damage. As found in the cluster analysis with all 16,390 genes, the pattern of gene expression using the smaller set of 1,811 IR-responsive genes revealed similarity between 24-hr post-IR and G_0_ cells, and differences with G_1_ cells (Figure 4B). One group of genes induced by IR at 24 hr was expressed at the highest level in G_0_ cells compared with G_1_-, S-, G_2_-, and M-synchronized cell populations (Zhou et al., unpublished observations) and asynchronous cell populations. Genes in this group included *GAS1* (growth-arrest–specific 1) that was first recognized as induced in G_0_ cells (Del Sal et al. 1992) and several p53 target genes, *FDXR*, *DDB2*, *BTG2*, *CCNG1*, *PA26*, and *SNK*. A second group of genes that was more highly expressed in G_1_ cells than in G_0_ and 24-hr post-IR cells included *G0S2*, *MYC*, *CCND1*, *CNK*, *ID1*, and *ID3*. The marked differences in gene expression patterns between G_1_ cells and G_0_ or 24-hr post-IR cells were largely contributed by these two groups of genes. Most of the cell cycle–regulated genes, functioning in the G_1_/S and G_2_/M transitions, were expressed at similar low levels in 24-hr post-IR, G_0_, and G_1_ groups compared with other asynchronous groups. The same set of 1,811 genes was used for three-dimensional PCA. The results with both asynchronous and synchronized cell populations showed that 24-hr post-IR components were very close to but did not overlap with the G_0_ components, and both 24-hr post-IR and G_0_ components were far from G_1_ components (Figure 4C).

## Discussion

### Cell cycle checkpoint functions and gene regulation in response to IR.

Although each cell line displayed a unique pattern of gene expression (Cheung et al. 2003), changes in gene expression related to cell cycle control, DNA metabolism, and apoptosis were very similar in the three lines in response to IR treatment. When DNA damage occurred in G_2_ cells (~ 10% of the total cell population), a stringent G_2_ arrest was observed 2 hr post-IR, as indicated by ≥ 94% reduction in mitotic cells. We did not expect the 1.5-Gy dose of IR to impede progress through and completion of mitosis. As a consequence of the IR-induced G_2_ arrest due to ATM- and ATR-dependent signaling (Deming et al. 2001; Kaufmann et al. 2003), the mitotic compartment emptied nearly fully. The expression of genes that controls the G_2_/M transition, particularly *Plk1*, *Cdc25C*, *CDC2*, and *cyclin B1*, was not changed at 2 hr post-IR, demonstrating the importance for this rapid G_2_ arrest of posttranscriptional modification of the above proteins. The ATM-CHK1 pathway responsible for changes in the phosphorylation status of CDC2 and Polo-like kinases has been well studied in activation of the immediate G_2_ checkpoint response post-IR (for a review, see Kaufmann et al. 2002). The G_2_ arrest induced by 1.5 Gy IR did not last long, however, because by 6 hr post-IR the frequency of mitotic cells in IR-treated cultures equaled or exceeded control values.

Synchronization of cells behind the G_1_ checkpoint can be expected to produce significant changes in gene expression. Given the approximately 4-fold reduction in S- and M-phase cells 24 hr post-IR as cells were arrested in G_1_, one would expect that the levels of expression of genes that are normally expressed in S, G_2_, and M phases would decline to a similar extent. More than 20 genes involved in cell cycle control and DNA metabolism were down-regulated by 4-fold or more 24 hr post-IR, implying that there was a common response to IR-induced DNA damage affecting large numbers of genes. The down-regulation of genes required for passage through S, G_2_, and M phases will be a necessary secondary consequence of prolonged accumulation of cells behind the G_1_ check-point. It is notable that the kinetic pattern of change in gene expression in diploid human fibroblasts given 1.5 Gy IR is quite different from that observed in HeLa cells given 10 Gy IR, where cells experience a prolonged G_2_ delay (Crawford and Piwnica-Worms 2001).

Genes that play important roles in the G_1_/S transition and initiation of DNA replication, including *CCNE*, *CDK2*, *CDC6*, *CDC45*, *CDC7*, *RFC* subunits, and *MCM* family members, were down-regulated at 6 and 24 hr post-IR as an expected consequence of the p53-dependent induction of *CDKN1A* (p21Waf1) to enforce the G_1_ checkpoint response. However, the p53/p21Waf1 pathway may not be the only one contributing to G_1_ arrest. For example, ARF (alternative reading frame) induced biphasic (G_1_ and G_2_) arrest in a p21-independent pathway (Modestou et al. 2001), and expression of c-Abl tyrosine kinase down-regulated the activity of CDK2 and induced G_1_ arrest in p21^−/−^ but not p53^−/−^ cells (Yuan et al. 1996). Many other p53 target genes (for early induced genes, see patterns 1 and 2 in Figure 3) may also play roles in p53-dependent cell cycle arrest. BTG2 induces accumulation of unphosphorylated RB by inhibiting cyclin D1 (Guardavaccaro et al. 2000). GADD45 stabilizes phosphorylation of serine-15 of p53 providing a positive feedback signal in activation of the p53 pathway (Jin et al. 2003), Plk3 phosphorylates p53 on serine-20 and enhances p53 stabilization (Xie et al. 2001), and p53-dependent activation of Plk2 prevents mitotic catastrophe after spindle damage (Burns et al. 2003). PPMD1, a member of the PP2C family of Ser/Thr protein phosphatases that is induced in a p53-dependent manner in response to various environmental stresses, reduces the phosphorylation of p53 through negative regulation of p38 MAP kinase, and in turn suppresses p53-mediated transcription and apoptosis (Bulavin et al. 2004). The p53-dependent G_1_ checkpoint orchestrates a complex signaling network to regulate the G_1_/S transition. Other p53-target genes seen in patterns 1 and 2, including *FDXR*, *PLAB*, *PA26*, *SES2*, and *TRF4*, may function in cell cycle regulation, although their correspondent functions have not been determined. However, at least one gene in pattern 2, *CCNG1*, has been reported to contribute to recovery of DNA synthesis after DNA damage through negative feedback regulation of p53 signaling (Ohtsuka et al. 2003). Several genes in pattern 1 have not been reported as p53-target genes (Bunz et al. 2002; Hoh et al. 2002; Sax and El-Deiry 2003; Yu et al. 1999). We found that several genes of unknown function, *I_1000314*, *OSBPL3*, *FLJ12484*, and *I_931541*, were regulated in a p53-dependent way upon DNA damage. These genes and most of the genes in patterns 1 and 2 were not induced by IR in fibroblasts expressing the HPV16E6 oncoprotein or a dominant-negative mutant p53 allele to inactivate p53 function (Zhou et al., unpublished observations).

### IR synchronized fibroblasts to a G_0_-like state.

An important finding in the present study was that 1.5 Gy IR induced a pattern of gene expression 24 hr post-IR that was more similar to G_0_ than to G_1_. Moreover, a G_0_-specific gene, *GAS1*, was markedly induced at 24 hr, indicating induction of a G_0_-like state of quiescence. Quantification of colony formation post-IR indicated that 40–45% of cells were permanently arrested and unable to form colonies. Although 55–60% of irradiated cells re-entered the cell cycle and successfully produced colonies, this resumption of DNA synthesis was not evident 24 hr post-IR. Di Leonardo et al. (1994) have charted the kinetics of DNA replication in irradiated fibroblasts and shown that a portion of cells arrested behind the G_1_ checkpoint resume DNA synthesis 24–72 hr post-IR. DNA double-strand breaks are thought to be the IR-induced lethal damage, and a small portion (2–5%) of DNA double-strand breaks is not rejoined by DNA repair (Dikomey et al. 2003). These unrepaired or misrepaired double-strand breaks likely account for an unremitting signal to arrest growth. Most of the observed 40–45% reduction of colony formation was considered previously to be a permanent G_1_ arrest (Di Leonardo et al. 1994; Walworth 2000). The present analysis of more than 16,000 genes showed that cells arrested behind the G_1_ checkpoint displayed a pattern of gene expression more reflective of G_0_ than of G_1_. This suggests that during prolonged IR-induced G_1_ arrest, additional reprogramming of gene expression occurs to bring cells into a G_0_-like state. To test for induction of replicative senescence, the senescence-associated marker pH 6.0 β-galactosidase (Dimri et al. 1995) was determined in a senescent fibroblast line (positive control) and in sham-treated or 1.5 Gy IR–treated cells from 1 to 7 days after the treatment. The percentage of β-galactosidase–positive cells was much higher in the senescent cells than in the sham- or IR-treated young cells, and no increased β-galactosidase staining was observed in IR-treated cells compared with sham-treated cells (data not shown). This result further supports the conclusion that IR induced a G_0_-like growth arrest instead of a senescence-like G_1_ arrest.

In summary, low-dose (1.5 Gy) IR induced marked G_1_ and G_2_ delays in normal human fibroblasts and caused stereotypic changes in the patterns of gene expression associated with these cell cycle delays. Early transcriptional activation of *CDKN1A* (*p21Waf1*) and many other p53-target genes as the effectors of G_1_ check-point response may account for the great majority of changes in gene expression recognized in irradiated fibroblasts. However, the sustained G_1_ arrest associated with induction of p21Waf1 in IR-damaged fibroblasts resembled a G_0_-like state of quiescence. In addition, direct transcriptional repression of important cell cycle–regulated genes through p53-dependent signaling may maintain the growth arrest. This aspect of DNA damage response will be addressed in a subsequent report.

## Figures and Tables

**Table 1:** DNA damage G_1_ and G_2_ checkpoint responses to 1.5 Gy IR (mean ± SD, *n* = 4–6).

Cell line	G_1_ arrest (%)^a^	G_2_ arrest (%)^b^
NHF1-hTERT	95 ± 2	97 ± 1
NHF3-hTERT	93 ± 2	95 ± 1
NHF10-hTERT	94 ± 1	94 ± 2

a G_1_ arrest was quantified by determining the percentage reduction in the fraction of cells in the first half of the S phase (Figure 2A).

b G_2_ arrest was quantified by determining the percentage reduction in the fraction of cells in mitosis (Figure 2A).

**Table 2:** EASE analysis of all genes selected by EPIG based on the Gene Ontology biological process.

Gene category	EASE score	Bonferroni^a^
Mitotic cell cycle	3.19 × 10^−33^	7.87 × 10^−30^
Cell cycle	4.33 × 10^−28^	1.07 × 10^−24^
Cell proliferation	5.35 × 10^−23^	1.32 × 10^−19^
DNA metabolism	1.59 × 10^−21^	3.93 × 10^−18^
DNA replication and chromosome cycle	6.16 × 10^−20^	1.52 × 10^−16^
M phase	2.92 × 10^−16^	7.20 × 10^−13^
Mitosis	3.35 × 10^−16^	8.24 × 10^−13^
M phase of mitotic cell cycle	7.48 × 10^−16^	1.84 × 10^−12^
Nuclear division	7.64 × 10^−16^	1.88 × 10^−12^
S phase of mitotic cell cycle	1.38 × 10^−15^	3.40 × 10^−12^
DNA replication	1.38 × 10^−15^	3.40 × 10^−12^
RNA metabolism	1.47 × 10^−13^	3.61 × 10^−10^
RNA processing	6.56 × 10^−13^	1.62 × 10^−09^
Regulation of cell cycle	6.87 × 10^−13^	1.69 × 10^−09^
Response to endogenous stimulus	1.49 × 10^−12^	3.68 × 10^−09^
Response to DNA damage stimulus	2.42 × 10^−12^	5.96 × 10^−09^
DNA repair	5.91 × 10^−12^	1.45 × 10^−08^
Cell cycle checkpoint	2.13 × 10^−10^	5.24 × 10^−07^
DNA-dependent DNA replication	4.35 × 10^−09^	1.07 × 10^−05^
Cell growth and/or maintenance	2.01 × 10^−08^	4.94 × 10^−05^
RNA splicing	2.18 × 10^−08^	5.36 × 10^−05^
mRNA processing	6.78 × 10^−08^	1.67 × 10^−04^
mRNA metabolism	2.04 × 10^−07^	5.02 × 10^−04^
RNA splicing, via transesterification reactions	2.24 × 10^−07^	5.52 × 10^−04^
Nuclear mRNA splicing, via spliceosome	2.24 × 10^−07^	5.52 × 10^−04^
Ribosome biogenesis and assembly	7.03 × 10^−07^	1.73 × 10^−03^
Ribosome biogenesis	8.23 × 10^−07^	2.03 × 10^−03^
rRNA processing	8.61 × 10^−07^	2.12 × 10^−03^
DNA recombination	1.31 × 10^−06^	3.23 × 10^−03^
rRNA metabolism	2.76 × 10^−06^	6.80 × 10^−03^
Cell organization and biogenesis	6.71 × 10^−06^	1.65 × 10^−02^
Regulation of mitosis	7.27 × 10^−06^	1.79 × 10^−02^

a *p*-Value adjusted for multiple comparison.

**Table 3:** EASE analysis of overrepresented genes in pattern 1.

Gene category	EASE score	Bonferroni
Negative regulation of cell proliferation	7.93 × 10^−06^	1.07 × 10^−03^
Regulation of cell proliferation	1.09 × 10^−04^	1.48 × 10^−02^
Regulation of cellular process	2.83 × 10^−04^	3.82 × 10^−02^
Regulation of biologic process	2.94 × 10^−04^	3.96 × 10^−02^
Cell proliferation	4.47 × 10^−04^	6.04 × 10^−02^
Regulation of cell cycle	6.12 × 10^−04^	8.26 × 10^−02^
Cell cycle arrest	1.42 × 10^−03^	1.91 × 10^−01^

## References

[b1-ehp0114-000553] Abraham RT (2003). Checkpoint signaling: epigenetic events sound the DNA strand-breaks alarm to the ATM protein kinase. Bioessays.

[b2-ehp0114-000553] Amundson SA, Bittner M, Meltzer P, Trent J, Fornace AJ (2001). Induction of gene expression as a monitor of exposure to ionizing radiation. Radiat Res.

[b3-ehp0114-000553] Arlett CF, Green MH, Priestley A, Harcourt SA, Mayne LV (1988). Comparative human cellular radiosensitivity. I: The effect of SV40 transformation and immortalisation on the gamma-irradiation survival of skin derived fibroblasts from normal individuals and from ataxia-telangiectasia patients and heterozygotes. Int J Radiat Biol.

[b4-ehp0114-000553] Bartek J, Lukas J (2001). Mammalian G_1_- and S-phase checkpoints in response to DNA damage. Curr Opin Cell Biol.

[b5-ehp0114-000553] Bishay K, Ory K, Lebeau J, Levalois C, Olivier MF, Chevillard S (2000). DNA damage-related gene expression as biomarkers to assess cellular response after gamma irradiation of a human lymphoblastoid cell line. Oncogene.

[b6-ehp0114-000553] Bulavin DV, Phillips C, Nannenga B, Timofeev O, Donehower LA, Anderson CW (2004). Inactivation of the Wip1 phosphatase inhibits mammary tumorigenesis through p38 MAPK-mediated activation of the p16(Ink4a)-p19(Arf) pathway. Nat Genet.

[b7-ehp0114-000553] Bunz F, Fauth C, Speicher MR, Dutriaux A, Sedivy JM, Kinzler KW (2002). Targeted inactivation of p53 in human cells does not result in aneuploidy. Cancer Res.

[b8-ehp0114-000553] Burns TF, Fei P, Scata KA, Dicker DT, El-Deiry WS (2003). Silencing of the novel p53 target gene Snk/Plk2 leads to mitotic catastrophe in paclitaxel (taxol)-exposed cells. Mol Cell Biol.

[b9-ehp0114-000553] Cheung VG, Conlin LK, Weber TM, Arcaro M, Jen KY, Morley M (2003). Natural variation in human gene expression assessed in lymphoblastoid cells. Nat Genet.

[b10-ehp0114-000553] Chou JW, Paules RS, Bushel PR (2005). Systematic variation normalization in microarray data to get gene expression comparison unbiased. J Bioinform Comput Biol.

[b11-ehp0114-000553] Cordeiro-Stone M, Boyer JC, Smith BA, Kaufmann WK (1986). Effect of benzo[*a*]pyrene-diol-epoxide-I on growth of nascent DNA in synchronized human fibroblasts. Carcinogenesis.

[b12-ehp0114-000553] Crawford DF, Piwnica-Worms H (2001). The G(2) DNA damage checkpoint delays expression of genes encoding mitotic regulators. J Biol Chem.

[b13-ehp0114-000553] Del Sal G, Ruaro ME, Philipson L, Schneider C (1992). The growth arrest-specific gene, gas1, is involved in growth suppression. Cell.

[b14-ehp0114-000553] Deming PB, Cistulli CA, Zhao H, Graves PR, Piwnica-Worms H, Paules RS (2001). The human decatenation checkpoint. Proc Natl Acad Sci USA.

[b15-ehp0114-000553] Dikomey E, Borgmann K, Brammer I, Kasten-Pisula U (2003). Molecular mechanisms of individual radiosensitivity studied in normal diploid human fibroblasts. Toxicology.

[b16-ehp0114-000553] Di Leonardo A, Linke SP, Clarkin K, Wahl GM (1994). DNA damage triggers a prolonged p53-dependent G_1_ arrest and long-term induction of Cip1 in normal human fibroblasts. Genes Dev.

[b17-ehp0114-000553] Dimri GP, Lee X, Basile G, Acosta M, Scott G, Roskelley C (1995). A biomarker that identifies senescent human cells in culture and in aging skin *in vivo*. Proc Natl Acad Sci USA.

[b18-ehp0114-000553] Doherty SC, McKeown SR, McKelvey-Martin V, Downes CS, Atala A, Yoo JJ (2003). Cell cycle checkpoint function in bladder cancer. J Natl Cancer Inst.

[b19-ehp0114-000553] Dulic V, Kaufmann WK, Wilson SJ, Tlsty TD, Lees E, Harper JW (1994). p53- dependent inhibition of cyclin-dependent kinase activities in human fibroblasts during radiation-induced G_1_ arrest. Cell.

[b20-ehp0114-000553] Eisen MB, Spellman PT, Brown PO, Botstein D (1998). Cluster analysis and display of genome-wide expression patterns. Proc Natl Acad Sci USA.

[b21-ehp0114-000553] Falck J, Mailand N, Syljuasen RG, Bartek J, Lukas J (2001). The ATM-Chk2-Cdc25A checkpoint pathway guards against radioresistant DNA synthesis. Nature.

[b22-ehp0114-000553] Fornace AJ, Amundson SA, Do KT, Meltzer P, Trent J, Bittner M (2002). Stress-gene induction by low-dose gamma irradiation. Mil Med.

[b23-ehp0114-000553] Guardavaccaro D, Corrente G, Covone F, Micheli L, D’Agnano I, Starace G (2000). Arrest of G(1)-S progression by the p53-inducible gene PC3 is Rb dependent and relies on the inhibition of cyclin D1 transcription. Mol Cell Biol.

[b24-ehp0114-000553] Hawse JR, DeAmicis-Tress C, Cowell TL, Kantorow M (2005). Identification of global gene expression differences between human lens epithelial and cortical fiber cells reveals specific genes and their associated pathways important for specialized lens cell functions. Mol Vis.

[b25-ehp0114-000553] Heffernan TP, Simpson DA, Frank AR, Heinloth AN, Paules RS, Cordeiro-Stone M (2002). An ATR- and Chk1-dependent S checkpoint inhibits replicon initiation following UVC-induced DNA damage. Mol Cell Biol.

[b26-ehp0114-000553] Heinloth AN, Shackelford RE, Innes CL, Bennett L, Li L, Amin RP (2003). ATM- dependent and -independent gene expression changes in response to oxidative stress, gamma irradiation, and UV irradiation. Radiat Res.

[b27-ehp0114-000553] Hoh J, Jin S, Parrado T, Edington J, Levine AJ, Ott J (2002). The p53MH algorithm and its application in detecting p53-responsive genes. Proc Natl Acad Sci USA.

[b28-ehp0114-000553] Jin S, Mazzacurati L, Zhu X, Tong T, Song Y, Shujuan S (2003). Gadd45a contributes to p53 stabilization in response to DNA damage. Oncogene.

[b29-ehp0114-000553] Kaufmann WK, Campbell CB, Simpson DA, Deming PB, Filatov L, Galloway DA (2002). Degradation of ATM-independent decatenation checkpoint function in human cells is secondary to inactivation of p53 and correlated with chromosomal destabilization. Cell Cycle.

[b30-ehp0114-000553] Kaufmann WK, Heffernan TP, Beaulieu LM, Doherty S, Frank AR, Zhou Y (2003). Caffeine and human DNA metabolism: the magic and the mystery. Mutat Res.

[b31-ehp0114-000553] Maher VM, Heflich RH, McCormick JJ (1981). Repair of DNA damage induced in human fibroblasts by -substituted aryl compounds. Natl Cancer Inst Monogr.

[b32-ehp0114-000553] Mendez J, Stillman B (2000). Chromatin association of human origin recognition complex, cdc6, and minichromosome maintenance proteins during the cell cycle: assembly of prereplication complexes in late mitosis. Mol Cell Biol.

[b33-ehp0114-000553] Modestou M, Puig-Antich V, Korgaonkar C, Eapen A, Quelle DE (2001). The alternative reading frame tumor suppressor inhibits growth through p21-dependent and p21-independent pathways. Cancer Res.

[b34-ehp0114-000553] Ohtsuka T, Jensen MR, Kim HG, Kim KT, Lee SW (2004). The negative role of cyclin G in ATM-dependent p53 activation. Oncogene.

[b35-ehp0114-000553] Ohtsuka T, Ryu H, Minamishima YA, Ryo A, Lee SW (2003). Modulation of p53 and p73 levels by cyclin G: implication of a negative feedback regulation. Oncogene.

[b36-ehp0114-000553] Sax JK, El-Deiry WS (2003). p53 downstream targets and chemosensitivity. Cell Death Differ.

[b37-ehp0114-000553] Slupphaug G, Kavli B, Krokan HE (2003). The interacting pathways for prevention and repair of oxidative DNA damage. Mutat Res.

[b38-ehp0114-000553] Stillman B (1996). Cell cycle control of DNA replication. Science.

[b39-ehp0114-000553] Unsal-Kacmaz K, Mullen TE, Kaufmann WK, Sancar A (2005). Coupling of human circadian and cell cycles by the timeless protein. Mol Cell Biol.

[b40-ehp0114-000553] Walworth NC (2000). Cell-cycle checkpoint kinases: checking in on the cell cycle. Curr Opin Cell Biol.

[b41-ehp0114-000553] Xie S, Wu H, Wang Q, Cogswell JP, Husain I, Conn C (2001). Plk3 functionally links DNA damage to cell cycle arrest and apoptosis at least in part via the p53 pathway. J Biol Chem.

[b42-ehp0114-000553] Yu J, Zhang L, Hwang PM, Rago C, Kinzler KW, Vogelstein B (1999). Identification and classification of p53-regulated genes. Proc Natl Acad Sci USA.

[b43-ehp0114-000553] Yuan ZM, Huang Y, Whang Y, Sawyers C, Weichselbaum R, Kharbanda S (1996). Role for c-Abl tyrosine kinase in growth arrest response to DNA damage. Nature.

